# Metabolic Regulation of Glial Phenotypes: Implications in Neuron–Glia Interactions and Neurological Disorders

**DOI:** 10.3389/fncel.2020.00020

**Published:** 2020-02-11

**Authors:** Ruqayya Afridi, Jong-Heon Kim, Md Habibur Rahman, Kyoungho Suk

**Affiliations:** BK21 Plus KNU Biomedical Convergence Program, Department of Pharmacology, Brain Science and Engineering Institute, School of Medicine, Kyungpook National University, Daegu, South Korea

**Keywords:** neuron, glia, metabolism, neuron–glia interaction, neurological disorders

## Abstract

Glial cells are multifunctional, non-neuronal components of the central nervous system with diverse phenotypes that have gained much attention for their close involvement in neuroinflammation and neurodegenerative diseases. Glial phenotypes are primarily characterized by their structural and functional changes in response to various stimuli, which can be either neuroprotective or neurotoxic. The reliance of neurons on glial cells is essential to fulfill the energy demands of the brain for its proper functioning. Moreover, the glial cells perform distinct functions to regulate their own metabolic activities, as well as work in close conjunction with neurons through various secreted signaling or guidance molecules, thereby constituting a complex network of neuron-glial interactions in health and disease. The emerging evidence suggests that, in disease conditions, the metabolic alterations in the glial cells can induce structural and functional changes together with neuronal dysfunction indicating the importance of neuron–glia interactions in the pathophysiology of neurological disorders. This review covers the recent developments that implicate the regulation of glial phenotypic changes and its consequences on neuron–glia interactions in neurological disorders. Finally, we discuss the possibilities and challenges of targeting glial metabolism as a strategy to treat neurological disorders.

## Introduction

The brain is composed of a complex network of neurons and glial cells carrying out the functions of the central and peripheral nervous system together (Silies and Klambt, 2011). Glial cells play diverse roles in maintaining the brain tissue homeostasis and neuronal integrity. The neurons are dependent on glial cells for energy provision, structural and functional support through the release of neuromodulators, axonal myelin formation, and the regulation of synaptic activity, as well as the surveillance of brain tissue microenvironment. Astrocytes and oligodendrocytes in close association with the vasculature are equipped with glycolytic machinery and efficiently supply nutrients and energy substrates from blood to the neurons and their long-extended axons (Belanger et al., 2011; Philips and Rothstein, 2017). Both astrocytes and oligodendrocytes sense the neuronal activity through glutamatergic signaling, which causes the increase in glucose uptake and provides the neurons and axons with lactate, hence coupling higher neuronal activity with energy supplies (Krasnow and Attwell, 2016; Steinman et al., 2016). On the other hand, microglia are brain resident immune cells, analogous to peripheral macrophages, that survey the brain tissue in their quiescent or resting state (Bachiller et al., 2018).

Neuroinflammation is the common denominator in many neurological disorders such as traumatic brain injuries and neurodegenerative diseases characterized by extensive structural and functional changes in the brain cells, including glial cells (Skaper et al., 2018). Glial cells are highly plastic and can undergo several changes ranging from proinflammatory neurotoxic to anti-inflammatory neuroprotective, that are collectively termed as phenotypic changes, in response to insult or injury to the brain (Suk, 2017). The glial phenotypic changes are characterized by morphological and functional changes, including high cellular reactivity and increased motility. Any insult or injury to the brain tissue is first sensed by microglia, which express receptors for a variety of ligands (Jha et al., 2019). The microglia adopt a neurotoxic phenotype characterized by an amoeboid shape in order to neutralize the invading culprit; however, the persistent insults observed in neurological disorders result in an exaggerated response leading to a state of chronic inflammation. The activated microglia release mediators that activate astrocytes and recruit oligodendrocyte progenitor cells to the site of inflammation or injury (Benarroch, 2013). The microglial phenotypic alteration is driven by a shift in its metabolic pathway, specifically, a reduction in the mitochondrial oxidative phosphorylation is reported in the activated microglial cells treated with an inflammatory stimulus (Nair et al., 2019). The activated microglia also play a significant role in the modulation of astrocytic functions, resulting in their neurotoxic activation that has detrimental effects on neuronal integrity during the course of the disease (Deng et al., 2010; Correa et al., 2011; Chen et al., 2015). Reactive astrocytes undergo several structural and functional changes, including metabolic modifications to maintain an optimum supply of lactate, ketone bodies, and glycogenolysis to release glucose, as well as mitochondrial deficits (Belanger et al., 2011). Astrocytic mitochondrial dysfunction has been reported in the amyotrophic lateral sclerosis (ALS) and neuroinflammation models, which show reduced mitochondrial coupling and oxygen consumption rates resulting in the neurotoxic activation of astrocytes and impaired neuronal function (Fiebig et al., 2019; Martinez-Palma et al., 2019).

The neuron-glia interactions are crucial for normal functioning of the brain during development and throughout adult life. The emerging line of evidence has shed light on the importance of glia in their bidirectional communication with neurons, their adaptability in various pathologies, modulation of neuronal activity, and phenotypic changes in response to neuronal injury (Benarroch, 2005; von Bernhardi et al., 2016). Throughout the brain tissue, neurons are closely associated with glial cells including astrocytes, oligodendrocytes, and microglia and their dynamic interactions are important for normal brain function (Chung et al., 2015; Meyer and Kaspar, 2017). It has been speculated that every type of neurological disease involves a glial component, which may be a primary or secondary cause (Kaminsky et al., 2016). Thus, the defensive and homeostatic abilities of glia specify their fundamental role in neuropathology.

The mechanisms regulating the various reactive states of glial cells are still elusive and can be attributed to the alteration in the metabolic profiles of these cells. The distinct metabolic changes coupled with mitochondrial alterations in the activated glial cells is gaining attention in the context of these phenotypic changes in neurological disorders (Benarroch, 2013). It has been reported that perturbations in the metabolic profile adopted by the glial cells also hamper the neuron-glial and inter-glial communications, resulting in an exacerbation of the ongoing reaction to the initial cause (Baik et al., 2019; Polyzos et al., 2019). Several reports highlighted the altered neuron-glial interactions in many neurological disorders (Pinto et al., 2018; Yamanaka and Komine, 2018). In this review, we describe the recent advances that examine the phenotypic changes driven by metabolic reprograming in glial cells and their consequences on neuron-glial interactions in various neurological disorders. Finally, the challenges and possibilities of targeting glial metabolism to modulate their phenotype are also discussed.

## Metabolic Regulation of Glial Phenotypic Changes

The brain consists of heterogeneous cells, and each cell has its own distinct metabolic profile. Among glial cells, this metabolic heterogeneity is more striking and is dependent on the tissue microenvironment surrounding the cells (Flowers et al., 2017; Barros and Weber, 2018). Multiple lines of evidence have demonstrated that astrocytes and oligodendrocytes are predominantly glycolytic, whereas microglia rely on oxidative phosphorylation for most of its cellular demands (Pellerin and Magistretti, 1994; Morrison et al., 2013; Holtman et al., 2015). Glial cells preferentially process glucose as a primary source for executing their cellular functions (Pellerin and Magistretti, 1994; Morrison et al., 2013; Holtman et al., 2015). These cells are endowed with glycolytic and mitochondrial machinery to process glucose for their own cellular demands as well as providing energetic support to neurons (Pellerin and Magistretti, 1994; Morrison et al., 2013; Holtman et al., 2015).

Most of the central nervous system (CNS) diseases induce a specific cellular response involving a cascade of reactions comprising complex interactions among a variety of brain cells. The duration and intensity of injury dictate the resulting cellular response, the persistence of which, results in a secondary pathological mechanism that causes more severe tissue damage. The response to brain injury is primarily mediated by glial cells, which become reactive to restore the brain homeostasis and to limit the damage. The phenomenon is referred to as reactive gliosis and is characterized by rapid alteration in the morphology, gene expression, as well as the secretome profile of glial cells, producing a broad effect on a myriad of cell types and functions (Henstridge et al., 2019). Astrocytes and microglia are major players in reactive gliosis and respond to a number of endogenous stimuli generated following injury, ischemia, as well as neurodegenerative diseases, such as multiple sclerosis (MS), Parkinson’s disease (PD), Alzheimer’s disease (AD), and ALS (Tezel et al., 2007; Allaman et al., 2010; Motori et al., 2013; Sun et al., 2013; McIntosh et al., 2019; Qiao et al., 2019; Wang et al., 2019).

A large body of literature has identified the neuroinflammatory responses in a variety of neurological disorders comprising of various glial phenotypes (Cherry et al., 2014). Both microglia and astrocytes can adopt proinflammatory (classical) or anti-inflammatory (alternative) phenotypes, depending on the invading stimuli and microenvironment. The microglial phenotypes identified as M1/M2 are adopted from the distinctive classification of the classical or alternative activation of macrophages, respectively (Nathan et al., 1983; Jha et al., 2016). The release of proinflammatory cytokines including TNF-α, IL-1β, IL-6, MIP-1α, and reactive oxygen/nitrogen species is attributed to M1 activation of microglia, whereas anti-inflammatory cytokines IL-10 and IL-4, as well as the upregulation of cell surface markers CD206 and Arg1, are the cardinal features of M2 microglia (Boche et al., 2013; Cherry et al., 2014). With the recent rise in glial research, this functional dichotomy of microglial polarization has now extended to astrocytes as well. Recent reports have identified that signal propagation from the distinct activation of microglia can also polarize the astrocytes (Liddelow et al., 2017). The proinflammatory or A1 and anti-inflammatory or A2 phenotypes of astrocytes were recently identified through *in vivo* and *in vitro* approaches, indicating that the neurotoxic activation of astrocytes can have detrimental effects in various disease scenarios (Liddelow et al., 2017). Besides the individual contribution of reactive astrocytes and microglia to various neurological pathologies, these reactive phenotypes also regulate the functions of oligodendrocyte during neuroinflammation. The underlying mechanisms driving these phenotypic changes have been attributed to multiple variables, such as the spatiotemporal position of these cells, specific pathology, as well as the aging process, which can influence the response of glial cells to the instigating stimuli (Radford et al., 2015; Grabert et al., 2016).

The evidence collectively highlights that alterations in the metabolic signatures of glial cells dictate their phenotypes, as well as their response to neuroinflammation (Edison et al., 2013; Jha et al., 2016). The functional dichotomy of astrocytic and microglial phenotypes is shown to be governed by their metabolic reprograming, more specifically, the alteration in glucose metabolism pathways, when activated by noxious stimuli. Recent studies have demonstrated the preferential increase in glycolysis by immune cells, similar to the Warburg phenomenon observed in tumor cells (Marelli-Berg et al., 2012; Yang and Chi, 2012). To optimize the production of adenosine triphosphate (ATP), the immune cells utilize glycolysis instead of mitochondrial oxidative phosphorylation to meet the increased cellular demands (Marelli-Berg et al., 2012; Yang and Chi, 2012). The AMP-activated protein kinase (AMPK) as well as phosphatidylinositol 3-kinase/protein kinase B (PI3K/Akt) signaling are believed to regulate the metabolic switch in the peripheral immune cells (Vats et al., 2006; Byles et al., 2013). The preferential increase in glycolysis over oxidative phosphorylation is well established in the peripheral immune cells including macrophages and regulatory T (Treg) cells, while there is still a major gap in knowledge regarding the metabolic reprograming in the context of reactive glial cells (Pearce and Pearce, 2013).

Microglia are equipped with efficient machinery for processing of a variety of biomolecules including glucose, ketone bodies, amino acids and free fatty acids (FFA), but glucose is the preferential substrate for energy production (Huang et al., 2018). Glucose is taken up through glucose transporter (GLUT) and metabolized to pyruvate, which then fuels the tricarboxylic acid cycle (TCA). Microglia express genes required for both oxidative phosphorylation and glycolysis, as revealed by transcriptomic analysis of mouse microglia (Zhang et al., 2014). Microglia metabolize FFA by lipoprotein lipase and synthesize acetyl-CoA through fatty acyl-CoA synthetase (Zhang et al., 2014). Microglia also take up glutamine through solute carrier transporter (SLC) receptors, which is then converted to α-ketoglutarate by mitochondrial enzyme glutamate dehydrogenase. The α-ketoglutarate in turn enters the TCA cycle for ATP production (Nakajima et al., 2015). Recently, it has been shown that GLT1 (glutamate transporter) expression is upregulated in microglia following inflammatory stimulation, indicating the possible role of microglia in glutamate recycling (Nakajima et al., 2015).

Under physiological conditions, microglia convert glucose to pyruvate, which is converted to ATP through mitochondrial oxidative phosphorylation. However, following activation, the microglial metabolism shifts from oxidative phosphorylation to glycolysis resulting in increased lactate production. Inflammatory stimulation of microglia with bacterial endotoxin lipopolysaccharide (LPS) or proinflammatory cytokines such as interferon-gamma (IFN-γ) results in a switch from oxidative phosphorylation to glycolysis (Gimeno-Bayon et al., 2014). Likewise, increased lactate production coupled with decreased ATP production and mitochondrial oxidative phosphorylation is reported in BV-2 mouse microglial cells stimulated with LPS (1 μg/mL, 3 h) (Voloboueva et al., 2013). The increase in glycolytic pathway in the microglial cells is also directly correlated with an increase in the expression of proinflammatory cytokines, indicating the association of metabolic reprograming with the neurotoxic activation of microglia. An increase in the release of nitric oxide (NO) was observed in BV-2 cells stimulated with LPS and IFN-γ, with concomitant potentiation of glycolysis confirmed by increased glucose consumption, lactate release, activity of hexokinase, glucose-6-phosphate dehydrogenase (G6PD), phosphofructokinase-1 (PFK1), as well as lactate dehydrogenase (LDH) activity (Gimeno-Bayon et al., 2014). In addition to the increase in enzymes involved in glycolysis in the activated microglia, a recent study also documented the increase in glucose transporter-1 (GLUT1) expression aiding the increased uptake of glucose by microglia when treated with LPS and IFN-γ (Wang et al., 2019).

Various signaling pathways are involved in the metabolic transition of M1 microglia. A recent report highlighted the possible effects of forkhead box P3 (FOXP3) in activated microglia and increase in the expression of glycolytic genes, such as lactate dehydrogenase A (LDHA) and hypoxia-inducible factor-1alpha (HIF-1α) (Sohn et al., 2012). FOXP3 is a well-known transcription factor that regulates the metabolic reprograming of Treg cells (Hori et al., 2003). The downregulation of FOXP3 in LPS-stimulated microglial cells resulted in increased expression of HIF-1α, LDHA, as well as NO, CXCL10, and MCP-1, implicating the repressive effects of FOXP3 on microglial transition through metabolic regulation (Chung et al., 2010). Moreover, recent reports identified the epigenetic regulation of glycolytic genes hexokinase 3 (HK3) and 6-phosphofructo-2-kinase/fructose-2,6-biphosphatase 3 (PFKFB3) by ten eleven translocation enzyme 2 (TET2) in neurotoxic microglia (Carrillo-Jimenez et al., 2019). TET2 regulates the inflammatory responses in bone marrow-derived macrophages and dendritic cells (Zhang et al., 2015; Cull et al., 2017). Inflammatory stimulation of the microglial cells increased the expression of TET2, inducing neurotoxic activation of microglia (Carrillo-Jimenez et al., 2019). The TET2-mediated transition is controlled by glycolytic enzyme PFKFB3 (Carrillo-Jimenez et al., 2019).

Unlike microglia, the astrocytes are inherently glycolytic cells, providing neurons with lactate as energy substrates. Astrocytes preferentially use glucose as a primary energy source. Astrocytes possess glycolytic and mitochondrial machinery to process glucose for their own demands and to provide energetic support to neurons (Mergenthaler et al., 2013). Glucose taken by astrocytes through GLUT1 is processed through glycolysis and mitochondrial oxidative phosphorylation (Baltan, 2015). Astrocytes also store glycogen and support neurons, when glucose is inadequately supplied by the blood (Mergenthaler et al., 2013). Moreover, astrocytes can also efficiently oxidize fatty acids, when brain supply of glucose is diminished (Ebert et al., 2003). The exact mechanisms of metabolic pathway acquisition in each glial cell in response to various stimuli are highly debated and need further exploration. Astrocytes have been showed to express various enzymes involved in fatty acid oxidation and ketogenesis, including carnitine palmitoyl transferase I (CPT1), mitochondrial 3-hydroxy-3-methyl glutaryl-CoA synthase (HMG-CoA), acetyl-CoA carboxylase (ACC), cAMP-dependent protein kinase (PKA) (Ebert et al., 2003). Indeed, astrocytes are the only cell type producing ketone bodies in brain, which serve as an important energy substrate for neurons during conditions of glucose and oxygen deprivation (Ebert et al., 2003). Astrocytic metabolic pathway, as any other cell types, is dictated by AMP/ATP ratio, neuronal activity and glycemic state (Ebert et al., 2003). Of note, astrocytes can also produce ketone bodies not only from fatty acid metabolism but also from amino acids (Auestad et al., 1991). It has been shown experimentally that the exposure of human astrocytes to recurrent low glucose conditions *in vitro* shifts their metabolism from glycolysis to fatty acid oxidation affecting the mitochondrial integrity (Weightman Potter et al., 2019).

To date, the metabolic profile of reactive astrocytes is not as fully understood as that of microglia. Astrocytes were considered as exclusively glycolytic cells with minimum reliance on mitochondrial oxidative phosphorylation (Walz and Mukerji, 1988; Pellerin and Magistretti, 1994, 2004). However, this concept has been recently challenged by numerous studies emphasizing the role of mitochondrial bioenergetics in astrocyte function (Cruz and Cerdan, 1999; Bluml et al., 2002; Bouzier-Sore et al., 2006). The presence of abundant mitochondria in the astrocytic fine processes implies the potential role of mitochondrial oxidative metabolism in addition to the glycolytic pathways, as a source of energy generation in astrocytes (Hertz et al., 2007; Lovatt et al., 2007; Mathiisen et al., 2010). The phenotypic changes observed in reactive astrocytes can be strongly associated with their metabolism, as seen in microglia, although further research is still needed to elucidate the clear definition of astrocyte metabolic reprograming induced by various stimuli. Transcriptomic analysis of reactive astrocytes in an ischemic brain injury model has revealed no significant changes in the glycolytic gene expression, however, few reports have highlighted the increased glycolytic activity in reactive astrocytes (Zamanian et al., 2012; Liddelow et al., 2017; Boisvert et al., 2018). To establish the role of metabolic reprograming in astrocytic phenotypic changes, the astrocytes were exposed to LPS and IFN-γ, resulting in an increased production of ATP through glycolysis coupled with reduced oxygen consumption rate, indicating the increase in glycolytic capacity and malfunctioning of mitochondria in the reactive astrocytes (Ferrick et al., 2008; Brand and Nicholls, 2011; Motori et al., 2013). Another study employing amyloid β-peptide (Aβ25-35) to stimulate astrocytes also showed similar results, i.e., increase in glycolysis and the release of lactate (Allaman et al., 2010). Moreover, HIF-1α and AMPK are reported to increase the expression of glycolytic genes in reactive astrocytes induced by NO released from the M1 microglia (Almeida et al., 2004; Brix et al., 2012).

Oligodendrocytes play a crucial role in regulating axonal metabolism. Neuronal axons are very long and require high energy during increased electrical activity, which cannot be supported by limited energy stores of neurons themselves: the energy requirements for ensuring the activity of Na^+^/K^+^ channels and for maintaining the firing of action potentials. Na^+^/K^+^ channels are abundantly expressed at the axonal internodes, and oligodendrocytes provide lactate through MCT1 transporter during high electrical activity (Ohno et al., 2011). Axonal injury was observed in organotypic spinal cord slice cultures, when MCT1 was inhibited, and addition of lactate to culture medium rescued the injury (Lee et al., 2012). Axonal injury in Mct1 heterozygous knockout mice precedes oligodendrocyte injury, indicating the importance of oligodendrocyte metabolic support to axons (Lee et al., 2012). Oligodendrocytes predominantly utilize glycolysis for ATP production, and are considered to fulfill high energy demands of axons by providing lactate, giving rise to neuron–glia compartmentation of brain metabolism. Oligodendrocytes are myelin-producing cells and require enormous amounts of energy for this purpose and hence utilize large amounts of metabolites such as lactate and glucose during development and remyelination after any demyelinating injury (Harris and Attwell, 2012). In myelinating oligodendrocytes, astrocytic-derived lactate is taken up through MCT1 transporters and converted to ATP through glycolysis or mitochondrial TCA cycle. Likewise, glucose can also enter oligodendrocytes through GLUT transporter for ATP generation. Moreover, oligodendrocytes utilize creatine as an energy source to fulfill their energy demands during active myelination. Creatine is synthesized endogenously in brain and enters oligodendrocytes through SLC6A8 (Chamberlain et al., 2017). The phosphate pool in the oligodendrocyte cytosol for ATP production is maintained by phosphocreatine, derived from creatine processing in the mitochondria.

The myelinating function of oligodendrocytes is tightly regulated by extracellular glucose concentration. It has been shown experimentally that low glucose results in the loss of myelinating potential of oligodendrocytes (Yan and Rivkees, 2006). Unlike astrocytes, oligodendrocytes cannot withstand energy deprivation for long time period; in fact, morphological changes of oligodendrocytes have been observed within 30 min after MCAO, and chromatin changes within 6 h (Pantoni et al., 1996). Oligodendrocyte progenitor cells (OPC) and oligodendrocytes show morphological changes under conditions of compromised metabolism (Zhou et al., 2018). Moreover, in metabolic deprivation states, as observed in ischemic conditions and MS, oligodendrocytes increase glycolytic metabolism for their own survival and lose myelinating functions. Loss of myelin, degeneration of distal oligodendrocyte process, and finally apoptosis is observed in MS lesion (Lassmann, 2016). MS pathogenesis is mainly attributed to failure of oligodendrocyte compensatory response to chronic metabolic failure, resulting in failure of remyelination. The functional and morphological changes in oligodendrocytes are not as evident as those of microglia and astrocytes. Also, oligodendrocyte changes are pathologic hallmark of demyelinating diseases. Nevertheless, exact metabolic pathways occurring in oligodendrocytes are still under extensive investigation.

## Altered Glial Metabolism in Neuron–Glia Communication and Neurological Disorders

The phenotypic polarization of microglia and astrocytes are identified in every neurological disease. Both astrocytes and microglia play a dual role in various diseases ranging from protective roles by disease resolution or neurotoxic roles by the release of mediators that potentiate the initial insult. Recent scientific advances point toward the alterations in the inherent metabolic machinery of reactive glial cells as a result of the excessive demands of the overall brain tissue, as well as the individual cellular demands in disease state, resulting in altered neuron-glial interactions. The gliotransmitters (e.g., ATP, glutamate, and D-serine) released by glial cells impact the neuron-glia communication (Osipova et al., 2018). It has been reported that impaired signaling between the microglia and neurons plays a significant role in the cognitive decline in AD, while the aberrant calcium and glutamate signaling in the reactive astrocytes also plays an important role in neuropathology (Teaktong et al., 2003; Lim et al., 2013). In ALS pathology, the neurotoxic activation of glial cells reduces the survival of motor neurons by purinergic and glutamate signaling leading to excitotoxicity (Haidet-Phillips et al., 2011). However, the bioenergetic mechanisms driving the neurotoxic or neuroprotective phenotype of glial cells in neurological diseases have still not been resolved and this area remains less explored (Table 1).

**TABLE 1:** Experimental findings related to metabolic alterations of glial cells.

Cell types	Stimuli/Models	Metabolic pathways	Suggested mechanisms	Remarks	References
BV-2 (mouse microglial cells)	LPS + IFN-γ	Enhanced glycolysis	Increased expression of glycolytic enzymes (PFK1 and LDH) Increased expression of glucose transporter (GLUT1 and GLUT4)	Increased glycolytic metabolism results in excessive lactate release from microglia, combined with increased oxidative stress	Gimeno-Bayon et al., 2014
BV-2 and B6M7 (microglial cells)	LPS + IFN-γ	Enhanced glycolysis	Increased expression of GLUT1 transporters	Increased neurotoxic activation of microglia and increased expression of IL-1β, TNF-α, CCL2, IL-12p40, and iNOS	Wang et al., 2019
Mouse primary microglial cell cultures	LPS	Enhanced glycolysis	Enhanced expression of HIF1-α and LDHA genes	FOXP3 is a key regulator of the metabolic reprograming of microglial cells following neurotoxic activation	Sohn et al., 2012
BV-2, CHME3, primary microglial cell cultures obtained from rats and mice	LPS	Enhanced glycolysis and reduced oxidative phosphorylation	Epigenetic regulation of glycolytic genes Hk3 and Pfkfb3 by TET2	Increased TET2 levels are reported in various neurodegenerative diseases and can be linked with the neurotoxic activation of microglia and the subsequent detrimental effects	Carrillo-Jimenez et al., 2019
Primary microglia cultures isolated from adult mice	Aβ_1__–__42_	Enhanced glycolysis and reduced oxidative phosphorylation	Not discussed	Glycolytic microglia have reduced phagocytic potential and increased inflammatory burden in AD pathology	Pan et al., 2019
BV-2 microglial cells expressing SOD1 G93A mutations	ALS	Reduced mitochondrial oxidative phosphorylation and increased glycolysis	Increased mitochondrial fragmentation induced by accumulation of mutant protein in microglial cells	Neurotoxic activation of microglia, propagating toxicity to astrocytes and neurons	Joshi et al., 2019
BV-2 microglial cells	Hypoxic environment (1% oxygen)	Increased glycolysis	Increased hexokinase 2 expression in microglia under hypoxic conditions leads to metabolic reprograming	Increased ROS and lactate released from hyper-glycolytic microglia exacerbate tissue damage in ischemic brain injuries	Li et al., 2018
Mouse primary microglial cell cultures	LPS	Inhibition of HDAC (repressor of glycolytic enzymes)	Increased M2 polarization (direct metabolic effects are not studied)	Increased ramification of microglia, increased phagocytosis, decreased release of proinflammatory cytokines	Huang et al., 2018
Primary astrocyte cultures obtained from mice	LPS + IFN-γ	Enhanced glycolysis and reduced oxidative phosphorylation	Altered mitochondrial dynamics leads to bioenergetic reprograming of astrocytes in an acute injury setting	Increased reactive oxygen species generation by metabolically reprogramed astrocytes	Motori et al., 2013
Primary astrocyte cultures obtained from mice	Aβ_25__–__35_	Enhanced glycolysis and other glucose consumption pathways	Aβ_25__–__35_ internalization increases the glucose uptake in reactive astrocytes	Metabolically reprogramed astrocytes are more neurotoxic to neurons, implying a possible role of reactive astrocytes in AD pathology	Allaman et al., 2010
Primary astrocyte cultures obtained from mice	NO	Enhanced glycolysis	HIF-1α drives the upregulation of enzymes involved in glycolysis	NO derived either from endogenous or exogenous sources, plays an important role in HIF-1α stabilization and regulation of glycolysis	Liu et al., 2015
hiPSC-derived astrocytes expressing *PSEN1*Δ*E9* mutation	AD	Increased oxidative phosphorylation	Mutations in astrocytes lead to disturbances in the glycolytic pathways	Increased reactive oxygen species generation, leading to oxidative burden in AD	Oksanen et al., 2017
Primary astrocytes isolated from SOD1^*G*93*A*^ transgenic rats	ALS	Compromised mitochondrial respiration and electron transport chain deficiencies	Accumulation of mutant SOD1 protein in astrocytes alters the metabolic machinery by mitochondrial dysfunction	Astrocytes bearing SOD1 mutations are more neurotoxic, leading to enhanced motor neuron deterioration in ALS pathology	Cassina et al., 2008
Aberrant glial cells obtained from spinal cords of symptomatic ALS rats	ALS	Reduced oxidative phosphorylation indicated by reduced mitochondrial respiration	Mitochondrial alterations are induced by mutant protein accumulation	Regulation of metabolic phenotype transition of glial cells in ALS appears to be an attractive strategy	Martinez-Palma et al., 2019
Primary astrocyte cultures isolated from rats	Pro-oxidant tert-butylhydroperoxide	Enhanced glycolysis and reduced oxidative phosphorylation	Oxidative stress induces astrocytic mitochondrial alterations	Increased lactate production and decreased ATP release negatively impact the tissue microenvironment after ischemic brain injuries	Almeida et al., 2012
Primary astrocyte culture isolated from mouse	Oxygen glucose deprivation	Upregulation of Sirt3 (enhanced oxidative phosphorylation)	Neurotoxic activation of astrocytes was inhibited	Inhibition of neurotoxic activation of astrocytes proves to be beneficial in ischemic brain injuries	Yang et al., 2017

### Neurodegenerative Disease

Neurodegenerative diseases such as AD, ALS, and Huntington’s disease (HD), include a wide array of age-related diseases with heterogeneous etiologies, and share the accumulation of neurotoxic mutant proteins, neurodegeneration with aging, oxidative stress, and neuroinflammation as their common signatures (Hroudova et al., 2014). The mutant protein accumulation, besides inducing the functional alterations in neurons, also induces the activation of glial cells. The glial cells have an important physiological role in maintaining brain homeostasis, and the loss of their function affects the trophic support in neurodegenerative diseases. Reactive glial cells induced by the accumulation of mutant protein in neurodegenerative diseases further contribute to impairment of the neuronal function by the increased release of proinflammatory cytokines, as well as the generation of reactive oxygen species.

AD is clinically characterized by a progressive decline in the cognitive ability and is pathologically associated with the presence of senile plaques consisting mainly of amyloid-β peptide (Aβ) and neurofibrillary tangles composed of hyperphosphorylated tau (Hroudova et al., 2014). An extensive body of literature has implicated impaired mitochondrial function, energy metabolism, and oxidative damage in the pathogenesis of AD (Ames, 2000). Marked gliosis, indicated by the presence of activated microglia and astrocytes around the Aβ plaques in the brain tissue is evident in AD pathophysiology (Nagele et al., 2003; Simpson et al., 2010; Cunningham, 2013). Aβ production and accumulation activates the glial cells, which potentiates the ongoing insult initiated by protein aggregates.

Glial cells play a very crucial role in phagocytosing Aβ aggregates; however, reactive glial cells have compromised phagocytic activity causing an increase in Aβ burden in the brain. Pan et al. (2019) recently demonstrated that increased glycolysis in the microglial cells was observed following treatment with Aβ peptide and resulted in reduced phagocytosis (Pan et al., 2019). When the metabolism of these cells was reprogramed toward oxidative phosphorylation, the cells restored their phagocytic activity (Pan et al., 2019). These results indicated the functional role of oxidative phosphorylation on the protective role of microglia (Pan et al., 2019). Moreover, the activated microglia augment the neurotoxicity of Aβ aggregates. Maezawa et al. (2011) reported the neurotoxic potential of microglial cells in co-cultures and hippocampal slices treated with 5–50 nM of amyloid-β oligomers (AβO) (Maezawa et al., 2011). The neurotoxicity was prevented when microglial activation was inhibited by doxycycline (Maezawa et al., 2011), indicating the neurotoxic potential of activated microglia in AD pathology. In a similar study, when deoxyglucose, an inhibitor of glycolysis, was added, it hampered the microglial neurotoxicity, which also pointed toward glycolysis as the primary energy reservoir for neurotoxic microglia.

Large amounts of Aβ were observed in the reactive astrocytes in human AD brain tissue, indicating an important role of these cells in Aβ clearance (Nagele et al., 2003). In addition, the severity of AD pathogenesis is positively correlated with Aβ aggregates in astrocytes and GFAP reactivity (Nagele et al., 2003; Olabarria et al., 2010; Simpson et al., 2010). A persistent neuroinflammatory environment combined with hypometabolism in the AD brain renders astrocytes with an altered metabolic activity to clear Aβ aggregates (Mrak et al., 1995; DeWitt et al., 1998). Thus, dysfunction of astrocyte metabolism is suggested to play a role in triggering the deposition of Aβ. As discussed earlier, astrocytes are inherently glycolytic and respond variably to various stimuli due to their metabolic plasticity.

In AD pathology, the astrocytes initially increase their glycolytic capacity to increase the clearance of Aβ aggregates, but as the disease progresses, the loss of several glycolytic enzymes reduces the phagocytic activity of astrocytes, resulting in neurodegeneration (Fu et al., 2015). In addition to Aβ deposition, an altered astrocytic metabolism also results in excessive ATP and glutamate release, which in turn leads to microglial activation and neurotoxicity (Orellana et al., 2011a, b). The metabolically dysfunctional astrocytes internalize Aβ aggregates and have been proven to be more neurotoxic in a co-culture model, and the neurotoxicity is mediated by the secreted factors from these astrocytes (Allaman et al., 2010). A study employing PSEN1 mutant iPSC-derived astrocytes showed that metabolic reprograming of astrocytes from glycolysis to oxidative phosphorylation increased reactive oxygen species and reduced lactate production (Oksanen et al., 2017). Hence, altered astrocytic metabolism can be correlated with increased oxidative stress and cognitive deficits in AD pathology (Oksanen et al., 2017).

Glial phenotypic transitions result in aberrant neuron–glial interactions, contributing to many clinical features of the CNS diseases including cognitive decline. Reactive astrocytes contribute to memory loss in AD through multiple pathways. Reactive astrocytes surrounding the Aβ plaques showed increased expression of multiple receptors and channels including nicotinic acetylcholine receptors α7nAChRs, A2A adenosine receptors, Ca^2+^-permeant ligand-gated channels, mGlu5 receptors, and P2Y1 receptors (Teaktong et al., 2003; Lim et al., 2013). Increased expression of these receptors and channels in the reactive astrocytes results in aberrant calcium (Ca2^+^) signaling and massive glutamate release leading to excitotoxicity (Lim et al., 2013).

Reactive astrocytes increase the tonic inhibition of dentate granule cells by releasing excessive GABA, which negatively impacts memory processing (Talantova et al., 2013). Increased glutamate release from the reactive astrocytes is known to cause increased synaptic loss by excessive neuronal *N*-methyl-D-aspartate (NMDA) receptor activation, thus augmenting the cognitive decline in AD (Talantova et al., 2013). Aβ-induced excessive activation of astrocytic α7nAChRs receptors activates extra-synaptic NMDA receptors (eNMDARs) leading to decreased frequency of miniature excitatory synaptic currents (mEPSC), increased NO production, and caspase 3 activation culminating in the loss of synaptic integrity (Talantova et al., 2013). Moreover, increased calcium oscillations in the reactive astrocytes lead to the opening of mitochondrial permeability transition pore (mPTP), potentiating the oxidative stress in neurons, as well as reducing the anti-oxidant glutathione (GSH) levels (Angelova and Abramov, 2014).

Neuronal accumulation of Aβ also alters the interaction of microglia with neurons by decreasing neuronal CD200 (Lyons et al., 2007a), a glycoprotein expressed on the neuronal cell surface, while microglia bear its receptor CD200R (Wright et al., 2000). Decreased neuronal expression of CD200 is observed in human AD brain, as well as the animal model of neuroinflammation, resulting in altered neuronal-microglial interactions (Lyons et al., 2007a). It has been recently shown that intracerebroventricular injection of IL-4 in rats enhanced the CD200-CD200R interaction, indicating the skewing of neurotoxic M1 microglia to neuroprotective M2 microglia. IL-4 administration also reduced release of proinflammatory cytokines from microglia and modulated long-term potentiation (LTP) in the AD model (Nolan et al., 2005; Lyons et al., 2007a). Enhancing the neuronal expression of CD200 also reduced neurotoxic activation of microglia, indicating the importance of bidirectional cross-talk between the two cells in maintaining brain homeostasis (Lyons et al., 2007a). Altered fractalkine signaling CX3CL1/CX3CR1 is the hallmark of AD pathology (Cho et al., 2011). Fractalkine (CX3CL1; FKN) is expressed mainly by neurons, and its receptor (CX3CR1) is expressed exclusively on microglia in the brain (Cho et al., 2011). In AD pathology, altered CX3CL1/CX3CR1 signaling between the neurons and microglia diminished synaptic activity and impaired cognitive functions due to fluctuations in long-term potentiation (LTP) (Simon et al., 2019).

The activated microglial and reactive astrocytes also constitute an important element of ALS, which is a late-onset neurodegenerative disease, involving the impaired survival of upper and lower motor neurons (Geser et al., 2008; Yamanaka et al., 2008; Gorter et al., 2019). Neurotoxic glial cells surrounding the motor neurons play a crucial role in ALS. It has been experimentally shown that the accumulation of mutant superoxide dismutase 1 (SOD1) in glial cells compromises their physiological functions and causes their neurotoxic activation.

Various recent reports have identified the induction of metabolic reprograming in glial cells by SOD1 mutations. Lower oxygen consumption rate (OCR) and reduced mitochondrial coupling state have been observed in neonatal astrocytes obtained from transgenic rats expressing human SOD1 mutation (SOD1-G93A) (Cassina et al., 2008). Motor neuron death induced by reactive astrocytes in ALS is mainly attributed to mitochondrial bioenergetic compromise in the astrocytes induced by SOD1 mutations (Vargas et al., 2006; Nagai et al., 2007). The reduced motoneuron survival by ALS-associated neurotoxic astrocytes is linked with its metabolic phenotype characterized by impaired mitochondrial metabolic pathways (Cassina et al., 2008). Reduced motoneuron loss and gliosis were also observed in the degenerating spinal cord of SOD1G93A rats, when treated with a metabolic modulator dichloroacetate (DCA) (Martinez-Palma et al., 2019). Moreover, the reduced toxicity of aberrant glial cells (AbGC) associated with ALS was observed when dichloroacetate (DCA) was added to the co-culture of astrocytes and motor neurons (Martinez-Palma et al., 2019).

In addition to the altered metabolic profile of astrocytes in ALS pathology, the neurotoxic microglia also cause damage to the neurons by amplifying the ongoing neuroinflammatory state (Appel et al., 2011). During the symptomatic stages of ALS, activated microglia are found in abundance in the spinal cords of SOD1-G93A mouse model (Appel et al., 2011). BV-2 microglial cells expressing the mutant SOD1-G93A showed altered oxidative phosphorylation, enhanced glycolysis combined with increased expression of neurotoxic proinflammatory cytokines (Joshi et al., 2019). These activated microglia also induced neurotoxic inflammatory astrocytes, as well as directly damaged the motor neurons, exacerbating the ongoing insult process (Joshi et al., 2019). Blunting the microglial neurotoxic activation in *in vivo* models of ALS has reduced the neurotoxicity and improved the survival of motor neurons. Inhibition of IL-1β, produced by activated microglia, increased the survival of mutant SOD1 animals (Roberts et al., 2013). Moreover, marked reduction in the neurotoxic profile of microglia was observed after the inhibition NF-κB in the co-culture system (Frakes et al., 2014). Modulating the glial phenotype via metabolic reprograming can thus be used to improve the clinical outcomes in ALS by reducing the direct neurotoxic potential of reactive glial cells.

The neuronal function is hampered in ALS by reactive glial cells via multiple pathways. The loss of trophic functions and gain of neurotoxicity by the glial cells have proven to be major drivers for the reduced survival of motor neurons in ALS (Haidet-Phillips et al., 2011). Increased release of gliotransmitters from the glial cells led to excitotoxicity augmented by increased oxidative burden (Wang et al., 2011). Dysregulation of the astrocytic neuromodulatory role contributed to neuronal excitotoxicity, depolarization, and hyperexcitability in ALS (Wang et al., 2011). Reduced number of astrocytic glutamate transporters resulted in dysfunctional glutamate cycling, leading to hyperexcitability of the motor neurons and subsequent death (Do-Ha et al., 2018). Aberrant calcium signaling and loss of lactate shuttling between the neurons and reactive astrocytes also propagate the disease severity (Madji Hounoum et al., 2017). In addition, increased transforming growth factor-β1 (TGF-β1) released by the neurotoxic astrocytes also compromised the neuroprotective role of microglia and infiltrating immune cells (Endo et al., 2015).

The loss of CX3CL1/CX3CR1 signaling induced by mutant protein accumulation halted the homeostatic roles of microglia, thus exacerbating the disease outcomes (Cardona et al., 2006). In transgenic mouse model of ALS, deletion of CX3CR1 led to aggravated neuronal loss (Cardona et al., 2006). Moreover, ATP produced by the damaged neurons potentiated the proinflammatory activation of microglia, resulting in the upregulation of chemotactic molecules and major histocompatibility class II molecules, leading to the recruitment of peripheral immune cells (Glass et al., 2010). The CD200 levels were found to be reduced in the frontal cortex of ALS patients, implying impaired microglia–neuron interactions (Umoh et al., 2018). Multiple studies have collectively shown that SOD1-induced microglial activation and neurotoxic astrocytes exerted more damage to the motor neurons by negatively impacting the cell-to-cell communication (Wang et al., 2011).

### Ischemic Brain Diseases

The neurological deficits and neurotoxic environment subsequent to ischemic brain injury is mainly mediated by the inflammatory processes initiated as a response to brain injury (Laterza et al., 2018; Zhang et al., 2019). Reactive gliosis plays a key role in the exacerbation of several pathological features of ischemic brain injuries (Laterza et al., 2018; Zhang et al., 2019). Recent advances in literature have established the reciprocal regulation between glucose metabolism and the pathological role of the reactive phenotype of microglia and astrocytes in various experimental settings for stroke and ischemia (Gaire et al., 2015; Ni et al., 2015).

Glial cells play a dual role in ischemic brain injury depending on the distinct phenotypic activation. It has been well-established that the anti-inflammatory glial cells are important to minimize the initial insult by releasing molecules and growth factors to aid in neoangiogenesis and functional recovery from the injury. The phenotypic transition and function vary in ischemic brain injury and depend on the regional and temporal distribution of glial cells.

Hyperglycolytic microglia are identified as the crucial drivers of neurotoxicity in ischemic brain injury settings, evident by the increased production of proinflammatory cytokines exerting neurotoxic effects. Hyperglycolytic microglia were identified in both *in vitro* and *in vivo* studies to increase the milieu of proinflammatory cytokines, increasing the damage to the brain tissue (Li et al., 2018). Reduction in the infarct volume and inflammatory cytokines were observed when hexokinase, a key enzyme in regulating glycolysis, was downregulated in the microglia by the employment of short hairpin RNA (shRNA) targeting HK2 (Li et al., 2018). A dual PPARα/γ agonist reduced the neurotoxic phenotype of microglia and the subsequent decrease in proinflammatory cytokines in the animal and cellular model of ischemic brain injury (Boujon et al., 2019). PPARα/γ are key regulators of genes controlling the expression of enzymes involved in glucose metabolism (Boujon et al., 2019). Although the study did not directly investigate the effect of PPARα/γ on microglial metabolism, it can be inferred that the modification of glucose metabolism in microglial cells may partly underlie the protective effect of the PPARα/γ agonist (Boujon et al., 2019). The protective effect of anti-inflammatory microglia after ischemic brain injury was exemplified by a recent report, which identified the upregulation of enzymes involved in oxidative phosphorylation and reduction in enzymes involved in glycolysis in the microglia cells isolated from the brain tissues treated with fractalkine (Lauro et al., 2019). Fractalkine treatment of animals subjected to permanent middle cerebral artery occlusion (pMCAO), polarized the microglial cells toward an anti-inflammatory phenotype by metabolic reprograming, resulting in reduction of injury, as well as accelerated repair (Lauro et al., 2019).

Reactive astrocytes are another large cellular fraction that perform various functions in ischemic brain pathology, depending on the polarized phenotype, as well as tissue microenvironment. Following ischemic and traumatic stroke, the reactive astrocytes are well recognized and known to form a protective scar limiting the further progression of damage. Also, these reactive astrocytes are important in the repair of the blood-brain barrier after injury (Sims and Yew, 2017). Increased GFAP expression after ischemic and traumatic brain injuries implies reactive astrogliosis, however, little is known about the phenotypic characteristics of these reactive astrocytes (Gleichman and Carmichael, 2014). In various traumatic and ischemic brain injuries, several functions of the reactive astrocytes have been identified ranging from protective to exacerbating inflammation by the excessive production of lactate, as well as proinflammatory cytokines (Li et al., 2017). Inhibition of reactive astrogliosis after ischemic brain injuries has been associated with improved clinical outcomes, such as reduction in the release of proinflammatory cytokines and reduced infarct volume (Li et al., 2017; He et al., 2018). Since ischemic brain injuries result in oxygen-glucose deprivation, the astrocytic glycolytic rate is first increased to compensate for high energy requirements in the injured brain. In contrast to cancer cells, where increased glycolysis is directly correlated with cytoprotection, in ischemic and traumatic brain injuries, the increased astrocytic glycolysis increased the ongoing acidosis by lactate release and accumulation. The increased acidosis ultimately hampers the neuronal integrity and functional outcomes of the disease (Dirnagl et al., 2009). Therefore, increasing the astrocytic oxidative phosphorylation may be neuroprotective and give better disease outcomes in ischemic brain diseases (Almeida et al., 2012). Moreover, traumatic and ischemic brain injuries impair neuron-glial metabolic coupling, evidenced by excessive lactate accumulation leading to cerebral metabolic failure. Since lactate is derived mainly from the glial compartment, it highlighted the increased astrocytic glycolysis following injury. There is deregulated shuttling of lactate between the astrocytes and neurons, contributing to rapid irreversible disease progression, as well as heightened neurotoxicity.

Taken together, it can be inferred from these findings that regulating the glial phenotypic transitions by metabolic modulation following traumatic and ischemic brain injuries might be exploited as novel therapeutic avenues (Lama et al., 2014).

### Demyelinating Diseases

Damage to the myelin sheath and axonal deterioration due to autoimmunity or other instigating stimuli are the common feature of demyelinating diseases encompassing several disorders, with MS being the most well-known. In MS pathology, the complex autoimmunity drives the disease pathogenesis, which involves a complicated crosstalk between the cells of the CNS and peripheral immune system. Neurotoxic activation of astrocytes and microglia is involved in the recruitment of autoreactive T-lymphocytes to the CNS by releasing chemotactic molecules. Inflammation-associated oxidative stress driving free radical-mediated tissue damage and demyelination is induced by the inflammatory activation of immune cells in MS (Fischer et al., 2012).

Several studies have examined the glycolytic shift of immune cells in MS (Angiari and O’Neill, 2018; Kornberg et al., 2018). In MS pathogenesis, increased glycolytic end products result in the production of oxidative stress, as well as increased release of proinflammatory cytokines, worsening the ongoing insult (Singh et al., 2001; Sternberg et al., 2018). In the brain tissue of MS patients, increased methylglyoxal, a glycolysis-derived product, was found in the reactive astrocytes and macrophages resulting in paracrine effects by activating their respective receptors on the microglia, increasing their inflammatory burden in disease (Wetzels et al., 2019). Moreover, increased expression of MCT4 and LDHA in astrocytes was also found in the brain tissues of MS patients and in experimental autoimmune encephalomyelitis (EAE), an animal model of MS (Kaushik et al., 2019), indicating the enhanced glycolytic metabolism of reactive astrocytes in MS.

The loss of myelinating potential of oligodendrocytes plays a central role in worsening the MS disease pathogenesis. It has been elucidated that the glycolytic activation of microglia in MS, not only increases the release of proinflammatory cytokines but also impairs the mitochondrial function and myelin gene expression in oligodendrocytes (Giri et al., 2018). Selective targeting of pyruvate dehydrogenase kinase 1 (PDK1) and Akt in microglia improved mitochondrial respiration and expression of myelin genes in oligodendrocytes (Giri et al., 2018). Collectively, these reports suggest that glial neurotoxic activation in MS not only increases the inflammatory burden but also impacts the neuron-glial metabolic coupling and the loss of myelinating potential of oligodendrocytes, thereby exacerbating disease pathogenesis (Liu et al., 2001; Zeis et al., 2015).

### Neuron-Glia Metabolic Interaction: Role of Glia-Secreted Metabolites

Neurons rely mostly on glial cells to meet their energy demands and the bioenergetic coupling between neurons and glial cells is crucial for brain functioning. Neuron-glia metabolic coupling is a complex phenomenon and involves a variety of enzymes carrying out the conversion of biomolecules, transporters responsible for shuttling of molecules between these cells, as well as cell surface receptors. Neurons require large amounts of ATP for executing their functions and maintaining mitochondrial integrity and membrane potential. Transport of nutrients and energy substrates to neuron is tightly regulated by neurovascular unit comprising endothelial cells, neurons, and glia. Endothelial cells express receptors for variety of metabolic substrates such as glucose transporters (GLUT1), monocarboxylate transporter for ketone bodies (MCT1 and 2), and CD36 for fatty acid (Yeh et al., 2008; Harjes et al., 2016). These transporters on endothelial cells act as a gateway to regulate the entrance of nutrients to the brain in a concentration-dependent manner. The nutrient supply to the brain depends on intercellular communication between astrocytes and endothelial cells. It has been recently shown that the NO released from the endothelial cells enhances glycolytic activity in astrocytes (San Martin et al., 2017). Astrocytic end feet which are in close contact with endothelial cells, thus becoming an active member of neurovascular unit, serve to protect neurons from oxidative damage, to release gliotransmitters, and to provide the source of energy substrates to neurons.

Astrocyte-neuron lactate shuttle (ANLS), which primarily focuses on astrocytic contributions to neuronal metabolic support, highlights the role of neuron-glial interactions in maintaining brain homeostasis (Genc et al., 2011). Besides astrocytes, recent studies have also identified the role of oligodendrocytes in supporting neurons metabolically, more specifically the axonal regions of the neurons (Genc et al., 2011; Lee et al., 2012). Both astrocytes and oligodendrocytes sense neuronal activity by the extracellular glutamate, released by neurons (Genc et al., 2011; Lee et al., 2012). The binding of glutamate to its respective receptors on both cell types increases the uptake of glucose through GLUT1. Inside the cells, glucose is either converted to pyruvate for oxidative phosphorylation by mitochondria or lactate through glycolysis. Astrocytes can also use intracellular glucose stored in the form of glycogen for production of energy. The lactate produced by astrocytes and oligodendrocytes can be actively shuttled to neurons through MCT transporters or it can be fueled back to pyruvate for ATP production or synthesis of fatty acids (Genc et al., 2011; Lee et al., 2012). Moreover, astrocytes play an important role in synaptic clearance of glutamate thereby preventing neuronal excitotoxicity (Genc et al., 2011; Lee et al., 2012). Inside the astrocytes, glutamate is converted to glutamine by glutamine synthase, which is transferred to neurons for synthesis of glutamate. There exists a close association among glutamate, glutamine, and TCA cycle metabolism in neurons, astrocytes, and oligodendrocytes (Genc et al., 2011; Lee et al., 2012). The metabolites released by glial cells are important for maintaining neuronal energy requirements, as neurons can utilize various substrates for ATP production, depending on various conditions such as fasting or during hyperactivity. Among the energy substrates, lactate is now accepted as a preferential source of ATP synthesis during hyperactivity (Bouzier-Sore et al., 2006). The lactate, which is mainly provided by astrocytes and oligodendrocytes, is known to play an important role in memory formation, as the inhibition of MCT2 in rat hippocampus resulted in memory impairment. The memory deficits were not rescued by injection of glucose, indicating the importance of glial-derived lactate in memory processing (Suzuki et al., 2011).

In addition to providing lactate, astrocytes also employ other mechanisms to regulate neuronal metabolism during hyperactivity. Increased neuronal activity during periods of information processing leads to generation of reactive oxygen species, which induces peroxidation of fatty acids existing as phospholipids in cellular membranes. Neurons have poor mitochondrial capacity to consume fatty acids for ATP production, and cannot make lipid droplets, therefore peroxidated fatty acids produced during high neuronal activity can lead to neurodegeneration (Schonfeld and Reiser, 2017). It has been recently shown that astrocytes are neuroprotective in periods of neuronal hyperactivity by taking up peroxidated fatty acids through lipoprotein particles and producing ATP by β-oxidation of fatty acids (Ioannou et al., 2019). The increased ROS production is counteracted by increased detoxification gene reaction (Ioannou et al., 2019). Moreover, astrocytic ATP increased in response to glutamate released from hyperactive neurons upregulates the activity of inhibitory interneurons, regulating excitotoxicity (Ioannou et al., 2019).

During neuronal hyperactivity, astrocytes also maintain synapse integrity and transmission through regulation of cholesterol and fatty acid synthesis. In astrocytes, cholesterol synthesis is regulated by sterol regulatory element binding proteins (SREBPs), which is abundantly expressed in hippocampal astrocytes (van Deijk et al., 2017). The deletion of SREBP cleavage-activating protein (SCAP) from astrocytes impaired secretion of cholesterol and phospholipids (van Deijk et al., 2017). Immature synapses were increased and presynaptic proteins were decreased in SCAP mutant mice, resulting in impaired short-term and long-term hippocampal synaptic plasticity (van Deijk et al., 2017). These results suggest the important role of cholesterol and fatty acid metabolism in the astrocytic control of neural activity.

## Modulation of Glial Metabolism as a Therapeutic Strategy

**FIGURE 1:**
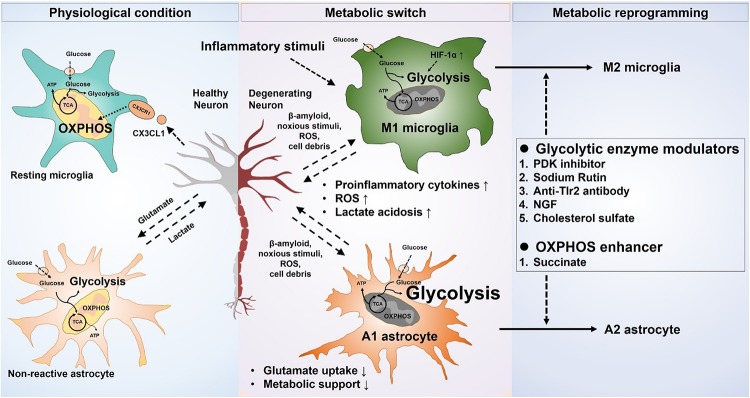
Metabolic modulation of glial phenotypes. Inflammatory stimuli induce neurotoxic glial phenotypes by increasing glycolysis and decreasing mitochondrial oxidative phosphorylation. Increased glycolysis results in an exaggerated release of proinflammatory cytokines, as well as oxidative burden. The proinflammatory cytokines released from the neurotoxic microglia can also propagate the signal to astrocytes, likewise, the neurotoxic astrocytic activation potentiates the neurotoxic microglia. Neuronal survival and integrity are negatively affected by the increased neuroinflammation, leading to a worse outcome in the neurological disorders. Selective targeting of glucose metabolism can modulate the glial phenotype toward a neuroprotective one and can improve the neuronal health in various disease scenarios.

The regulation of phenotypic polarization of the macrophages and T-lymphocytes by concomitant reprograming of the metabolic pathways is a well-established phenomenon. Recent advances in immunometabolism have identified many drug targets and putative candidates to reprogram the macrophages, as well as the effector and regulatory population of T cells in various inflammatory and autoimmune diseases (Kelly and O’Neill, 2015; Patsoukis et al., 2015). Metabolic reprograming of glial cells to fulfill their own cellular needs, as well as for the execution of their effector roles in various disease conditions, has recently gained attention. Due to the complexity of the underlying metabolic pathways and the lack of definitive targets regulating the glial metabolism, very few studies have examined glial metabolic modulation. The results of these studies indicate the importance of phenotype-specific modulation of glial cells compared to blocking of activation as being more beneficial in disease conditions (Figure 1).

Mounting evidence suggests the protective role of enhancing glial oxidative phosphorylation and the resultant reduction in the expression of neurotoxic proinflammatory cytokines in various disease settings. Recently, the neuroprotective effects of a flavonoid sodium rutin in AD model were attributed to enhanced oxidative phosphorylation in the microglial cells (Pan et al., 2019). Enhancing the oxidative phosphorylation in microglial cells reduced the amyloid beta burden and improved memory deficits (Pan et al., 2019). In line with these results, treatment of LPS/Aβ-stimulated primary microglial cells with anti-TLR2 antibody inhibited the expression of glycolytic enzyme (PFKFB3), as well as reduced the proinflammatory cytokine release and enhanced the phagocytic ability (Rubio-Araiz et al., 2018). Neurotrophins and nerve growth factor (NGF) inhibited LPS-stimulated primary microglial responses by downregulating the glycolytic enzymes Pfkβ3 and Ldhα (Fodelianaki et al., 2019).

Increased mitochondrial oxidative phosphorylation in primary astrocyte cultures following cholesterol sulfate treatment enhanced neuroprotective potential of astrocytes (Prah et al., 2019). The same study also found decreased phosphorylation of AMPK in astrocytes, which is a key regulator of glycolysis. Astakhova et al. (2019) recently showed increased AMPK-mediated expression of prostaglandin endoperoxide synthase 2 (Ptgs2), which regulates the expression of prostaglandins and eicosanoids (Astakhova et al., 2019). Inhibition of mitochondrial respiratory chain complexes in the astrocytes increased the mRNA expression of inflammatory genes (Astakhova et al., 2019). The stimulation of astrocytes *in vitro* with TNF-α and IL-1α augmented the inflammatory response following pharmacological inhibition of mitochondrial oxidative respiration (Astakhova et al., 2019).

Ketogenic diet has been shown to exert neuroprotective effects in a variety of modalities, principally modulating neuronal metabolism (Simeone et al., 2018). Ketone bodies play an important role in epileptic seizure by regulating calcium signaling (Simeone et al., 2018). Recently, it was shown that ketone bodies also inhibit inflammatory activation of microglia by regulating histone deacetylases (HDACs), which are transcriptional repressors of genes regulating glycolytic enzymes (Das Gupta et al., 2016; Huang et al., 2018). LPS-induced inflammatory activation of BV-2 microglial cells was inhibited by treatment with ketone bodies, which was attributed to inhibition of HDAC (Huang et al., 2018). Another evidence of epigenetic regulation of microglial metabolism comes from the study evaluating the anti-inflammatory activity of sirtuin 3 (SIRT3) (Cao et al., 2019). Silent mating-type information regulator 2 homolog (SIRTs) are known to regulate the genes responsible for metabolic enzymes (Finley and Haigis, 2012). SIRT3 reduced the production of superoxide dismutase and increased the migratory potential of microglia cells *in vitro* (Cao et al., 2019). Upregulation of SIRT3 in astrocytes also inhibited their inflammatory activation, highlighting the possibility of metabolic regulation by targeting epigenetics (Yang et al., 2017). Adjudin treatment promoted the functional recovery in ischemic mice by inhibiting the inflammatory activation of astrocytes through Sirt 3 upregulation (Yang et al., 2017).

Succinate, an intermediate of TCA cycle, has shown promising effects in patients with traumatic brain injury. Its protective effects are mediated by enhancing the ATP production through mitochondrial oxidative phosphorylation (Giorgi-Coll et al., 2017). Mixed glial cells under metabolic stress induced by rotenone showed increased lactate/pyruvate ratio (LPR) as well as mitochondrial deficits (Giorgi-Coll et al., 2017). Mitochondrial dysfunction in the glial cells was rescued after 24 h treatment with succinate, indicating the possible role of glial mitochondrial metabolism as a target of intervention in ischemic and traumatic brain injuries (Giorgi-Coll et al., 2017). DCA is a pyruvate structural analog and inhibitor of PDKs, thereby inhibiting the phosphorylation of pyruvate dehydrogenase (PDH). Several studies have reported the protective effects of DCA in various neurological disorders including gliomas, ischemic brain diseases, as well as neurodegenerative diseases. It has been reported that DCA treatment of aberrant glial cells (AbGC) derived from the spinal cords of SOD1-G93A rats reduced their neurotoxic activation, extracellular lactate accumulation, and improved mitochondrial function (Martinez-Palma et al., 2019). The survival and function of motor neurons were also improved when co-cultured with AbGC in the presence of DCA (Martinez-Palma et al., 2019). Moreover, *in vivo* administration of DCA to ALS rats also improved the functional outcomes and reduced gliosis (Martinez-Palma et al., 2019). DCA treatment also reduced the activation of microglia and astrocytes in glucose deprivation-induced injury in rats (Kho et al., 2019). These studies highlight the metabolic profile of glial cells as possible targets for intervention in treating ALS and ischemic brain injuries.

## Conclusion and Future Perspective

It has now been clearly identified that neuroinflammation heavily influences the pathology of many neurological disorders. The therapeutic interventions modulating the glial functions in ischemic, demyelinating, and neurodegenerative diseases have shown promising results in animal and preclinical studies. Glial phenotype polarization has been defined in the *in vitro* and *in vivo* settings with the help of recent technical advances in glial research, and distinctive markers for each subpopulation of polarized glial cells have also been identified. The mechanism driving the circumstantial activation of glial phenotypes is just starting to unravel. Intensive research is required to clearly demarcate the regional and temporal transitions of the glial cells in various clinical scenarios and to allow the identification of putative targets to skew this transition toward a neuroprotective phenotype.

The crucial findings described above highlight the role of metabolism in driving the phenotypic transition of glial cells in various neurological disorders and necessitate further investigation to enhance the supportive functions of these cells through metabolic modulation. Many recent studies have highlighted the role of enhanced glycolysis in neurotoxic activation of microglia, resulting in increased release of proinflammatory cytokines and reduced phagocytosis (Lyons et al., 2007b; Zhang et al., 2015; Lauro et al., 2019; Wang et al., 2019). On the contrary, few studies also reported that enhanced glycolysis in microglia augments their phagocytic activity to reduce the accumulation of protein aggregates in neurodegenerative diseases. A recent study showed that the inflammatory activation of microglia with IFN-γ increases phagocytosis of Aβ aggregates (Baik et al., 2019). However, another study suggested that the phagocytic function of microglia is enhanced when mitochondrial oxidative phosphorylation was upregulated (Pan et al., 2019). Henceforth, the contrasting findings of various studies necessitate in-depth investigation to clearly delineate the role of metabolic programing in various activation states and phenotypes of microglia.

The intimate association of glia with neuronal cells and the consequent effect on neurons is evident by the growing wealth of evidence. Several studies have highlighted that metabolic perturbations of glial cells altered neuron-glial interactions, potentiating the underlying pathology of many neurological diseases. Preliminary studies targeting the glial metabolism in neurodegenerative, ischemic brain injuries, and demyelinating disorders showed decreased oxidative burden, reduced production of proinflammatory cytokines, and reduced damage to neurons. Therefore, future studies unraveling the underlying pathways regulating the metabolic alterations in reactive glial cells will open a new frontier for the development of novel therapeutic modalities.

## Author Contributions

All authors listed have made a substantial, direct and intellectual contribution to the work, and approved it for publication. RA, J-HK, and KS formulated the focus of this review. J-HK and MR conducted the literature review and participated in the discussion. RA and KS wrote the manuscript.

## Conflict of Interest

The authors declare that the research was conducted in the absence of any commercial or financial relationships that could be construed as a potential conflict of interest.
